# An Active Inference Agent for Modeling Human Translation Processes

**DOI:** 10.3390/e26080616

**Published:** 2024-07-23

**Authors:** Michael Carl

**Affiliations:** Department of Modern and Classical Language Studies, Kent State University, Kent, OH 44240, USA; mcarl6@kent.edu

**Keywords:** active inference, predictive processing, translation process research, translation agent

## Abstract

This paper develops an outline for a hierarchically embedded architecture of an artificial agent that models human translation processes based on principles of active inference (AIF) and predictive processing (PP). AIF and PP posit that the mind constructs a model of the environment which guides behavior by continually generating and integrating predictions and sensory input. The proposed model of the translation agent consists of three processing strata: a sensorimotor layer, a cognitive layer, and a phenomenal layer. Each layer consists of a network of states and transitions that interact on different time scales. Following the AIF framework, states are conditioned on observations which may originate from the environment and/or the embedded processing layer, while transitions between states are conditioned on actions that implement plans to optimize goal-oriented behavior. The AIF agent aims at simulating the variation in translational behavior under various conditions and to facilitate investigating the underlying mental mechanisms. It provides a novel framework for generating and testing new hypotheses of the translating mind.

## 1. Introduction

Translation has many definitions. It has been defined as the “process of creating target-language content that corresponds to the source content according to agreed-upon specifications” ([1], p. 7), or/and it “has to be an accurate reproduction of the source text in a target language” ([2], p. 94). It has been defined as a process and a product [3] requiring the integration of linguistic, cognitive, and cultural knowledge. Despite the variety of definitions, it is uncontroversial that human translators demonstrate remarkable proficiency in navigating this process, but understanding the underlying mental mechanisms remains a challenging endeavor. Numerous models have been proposed [4,5,6,7,8,9,10,11,12,13,14], as frameworks for elucidating processes in translation, but they are often purely pedagogical or are otherwise unsuited approaches for empirical assessment or verification. However, in recent years, models based on active inference (AIF, [15,16]) and predictive processing (PP, [17,18,19]) have emerged as promising frameworks for modeling and simulating complex mental processes that may also be suited for modeling human translation processes [20]. These theories propose that the mind constructs a model of the environment, continually generating and integrating predictions and sensory input to guide behavior. Leveraging these theoretical foundations, this paper elaborates on a novel architecture for an artificial agent aimed at modeling human translation processes. The architecture comprises three embedded processing strata—a sensorimotor, a cognitive, and a phenomenal layer—where each layer represents a different aspect of the translating mind. Each layer consists of a structure of states and transitions that are conditioned on observations of the environment and/or the other processing layers, while transitions between the states are conditioned on actions that follow plans to minimize expected free energy [15,16] and optimize goal-oriented behavior. The proposed AIF agent aims at simulating translation behavior and opens new possibilities for investigating the underlying mental mechanisms governing this complex mental task. 

This paper thus contributes to the growing body of research that elicits our understanding of human translation processes. It proposes a novel way of approaching translation research through the simulation of the temporal structure in which humans produce translations. In order to appreciate the current situation of Cognitive Translation and Interpretation Studies (CTIS) and how the proposed AIF agent may enrich the CTIS research landscape, Section 2 provides a brief historical review of 40 years of empirical translation research, outlining how this field of enquiry has increasingly endorsed embodied and enactive approaches in the past years. The multitude of theoretical and methodological approaches has led to several sub-denominations in the field, all of which can, however, be subsumed under the CTIS label. 

In Section 3, I suggest that three distinct mechanisms of primed anticipation are essential in human translation processes: perceptual/semantic priming unfolds on the sensorimotor layer, conceptual/associative priming processes unwind on the cognitive layer, and affective priming on the phenomenal layer. The pausing structure in translation production, I argue, is tightly related to the priming strength (or the lack thereof), where stronger priming effects will likely result in more immediate translation production and thus shorter inter-keystroke pauses. 

In Section 4, I discuss two different approaches for conceptualizing keystroke pauses, a much-discussed topic in empirical translation research [21,22,23]. I illustrate the relationship between the Task Segment Framework (TSF, [22]) and the HOF taxonomy [23] by means of a translation progression graph. While Section 4 provides evidence for hierarchically embedded pausing structures in translation production from a conceptual view, Section 5 and Section 6 aim lay out an architecture for the artificial AIF translation agent suited to simulate the pausing structure in human translation production. Section 6 maps the pause structure, as observed in the translation process data discussed in Section 4, on the suggested embedded hierarchical architecture. 

Section 7 and Section 8 provide additional empirical support for the suggested AIF architecture. These sections discuss the variation in typing behavior, pauses and Tasks, as observed in recorded behavioral data from 54 Spanish and Arabic translators. It discusses the recorded process data in light of the TSF and HOF states and the proposed AIF architecture. While Section 7 is concerned with keystroke pauses, Section 8 assesses properties of the translator’s typing bursts (Tasks). This paper ends with a discussion in Section 9 which provides a wider outlook on some topics currently discussed within the CTIS literature and elaborates novel views on human translation processes that emerge from different interaction possibilities between the three processing layers of the AIF agent and its implementation as embedded Partially Observable Markov Decision Processes (POMDPs, Heins, 2022 [24]).

## 2. A Brief Review of CTIS 

Cognitive Translation and Interpretation Studies (CTIS) is a field of study within translation studies that has a relatively short history. CTIS has emerged in recent years as a research discipline that encompasses several research traditions, aiming to understand cognitive processes of translators, including human–computer interaction, emotions, or workplace ergonomics. The first attempts to study translation as a cognitive activity date back to the 1960s and 1970s. This early research was targeted to investigate spoken translation and interpretation [5]. By the mid-1980s, translation process research (TPR) started investigating the mental processes of written translation, Machine Translation post-editing, and translation revision, using think aloud protocols (TAP). In the mid-1990s, with the wide-spread availability of Personal Computers and the development of Translog [25], it became possible to record the translators’ keystrokes, introducing a new era of quantitative investigation. The TRAP (translation process) project was the first endeavor in this regard:

*“[R]esearchers from different language departments at the Copenhagen Business School (CBS) launched a translation project with the aim of promoting research into the translation process … [as] it was felt that our understanding of the mental processes could be improved if the traditional qualitative approaches could be supplemented by quantitative data.”*

([26], p. 7)

Keylogging technology, and from 2005 also eyetracking [27], provided the empirical basis for TPR to investigate and model the workings of the translators’ minds. TPR thus set out to investigate how translators translate, how they create meaning, how they arrive at translation choices, how translation competence develops, how cultural and linguistic factors impact the translated text, etc. (see, e.g., [28]), with the ambition to assess “by what observable and presumed mental processes do translators arrive at their translations?” ([29], p. 21). By around 2009, the first attempts were made to collect translation process data [30] in a centralized repository that was to become the CRITT TPR-DB (Translation Process Research Database, [31]) of the Center for Research and Innovation in Translation and Translation Technology (CRITT).

Based on recorded keylogging, and in many cases also eyetracking data, TPR has investigated several theoretical frameworks to explain behavioral observations: Hvelplund, 2011 [32] draws on working memory and the central executive system [33] to explain translation processes, while Sjørup, 2013 [34] assesses the cognitive effort in metaphor translation referring to the work of [35]. Other researchers investigate predictions of systemic functional and cognitive grammar for understanding and explaining translation processes [36,37]). Numerous researchers develop measures of cross-lingual complexity on a lexical or syntactic level [38,39,40], while Alves and Vale, 2009 [41] develop an empirical interpretation of translation units (TUs, see below) to ground Relevance Theory (RT, [10,42]) in empirical behavioral data. Schaeffer and Carl, 2013/2015 [14] suggest a Monitor Model that builds on the findings of Tirkkonen-Condit, 2005 [43], which “hypothesized literal translation automaton and its monitoring mechanism”, as well as aspects of bilingualism [44,45]. Further work [46] joins the Monitor Model with insights from RT and AIF [20].

However, as Risku, 2012 [28] (p. 675) points out, earlier TPR researchers “refer to and expand” the classical computational model of cognition, in which the “mind is seen essentially as a problem-solving mechanism for information-processing” ([47], p. 3). Subsequently, several post-cognitivist approaches have emerged, subsumed within CTIS, that account for the embodied, embedded, extended, enacted, and affective (4EA) nature of translation [2,48,49,50,51].

In 2010, Muñoz [48] called for an alternative denomination within empirical CTIS which he labeled *cognitive translatology*. In his foundational paper, Muñoz elaborates upon ten suggestions for a functionalist, cognitive translatology program with the “urgent need to establish experimental paradigms to foster the interplay between theory and research” (p. 169). He seems to endorse “a change of focus to study the dynamics of the interaction among mind, body, and the environment” and a view of cognition that holds that “what is going on inside the head, is not adequate to describe the workings of the mind” (p. 171). Muñoz [48] posits that translators “enter into deeper mental processing strategies, namely, problem-solving and decision-taking, only when direct, proceduralized formulations do not seem successful” (p. 177). Cognitive processes, he says, “happen in real-time environments and cognitive agents adapt and react to environmental challenges” (p. 171). Cognitive translatology, therefore, “should focus on the interaction between translators and their environment” (p. 178).

Other translation scholars follow similar approaches. Risku, 2014 [52] for instance, makes out a methodological shift in the theoretical orientation and proposes conducting research at the workplace to complement TPR with “qualitative, ethnographic research in order to be able to account for the situated, embedded and extended aspects of cognition” (p. 331). She attributes thereby “a central role in human cognition to the body and to physical and social interaction rather than to the notion of mental representation” ([49], p. 481). Similarly, Ehrensberger-Dow, 2017, 2021 [53,54] investigates how workspace “ergonomics can provide insights into the physical, cognitive, and organizational factors that impinge on translation” (2017: p. 332), while O’Brien, 2017 [55] and many other researchers are interested in Machine Translation post-editing and the efficiency of post-editing processes.

Another line of research focuses on emotional intelligence in translation. For Hubscher-Davidson, 2017 [56] emotional intelligence not only plays an important role in job satisfaction but also makes translators more skilled in dealing with emotion-laden and difficult issues in their work. Hubscher-Davidson, 2017 [56] (p. 2) defines “emotionality as a set of responses that are observable or perceivable to us or to others around us and take place when a person reacts emotionally to stimuli”. She sees “three distinctive areas where emotions influence translators: emotional material contained in source texts, their own emotions, and the emotions of source and target readers”. For Hubscher-Davidson and Lehr, 2021 [57] “affective profiles of translators can sometimes be more important than their language skills in terms of shaping translations” (p. 18). Consequently, “Translators who are aware of their own dispositions and emotional processing styles will be in a better position to make effective decisions at work” ([57], p. 19). This view is shared, for instance, by Rojo, 2017 [58], who maintains that emotional intelligence and intuition are essential in translation performance.

This embodied/affective position is further developed, for instance, by Robinson, 2023 [2]. Drawing on Peirce (1992) [59], Robinson distinguishes between three kinds of “interpretants”: the “emotional”, the “energetic”, and the “logical” interpretants. He argues that feelings are qualia, and qualia are emotional interpretants. Emotional interpretants are activated first, followed by energetic and cognitive interpretants: “we move from feeling-as-First to effort-as-Second to thought-as Third” (p. 31). In this view, we first “feel the word” (ibid.), then we expend effort to “put the word into some kind of experiential context” (i.e., the energetic interpretant), and then we “construct the word’s meaning out of the clash of the emotional and energetic interpretants”. For him, every word and every phrase are “*constantly being enactively and iteratively generated by human interpretive consciousness cycling through emotional and energetic interpretants up to the verbalizations of logical interpretants*” (p. 38, original emphasis). Emotions, he says, have thereby a social status; they are “performative responses to other people, embodied addresses signaling not only what I’m thinking about what you just did/said but how my thinking and feeling aligns with and/or deviates from yours/ours” (p. 63). Emotions are internal to an agent, but they also surface though body language, mimetic, and nonverbal communication. Affect, he says, “is the glue that makes the world we cocreate with our environments cohere” (p. 86). This is what Robinson calls *the somatic exchange*: affect is not trapped in the individual body. Rather, he maintains, it is the “extensibility of feeling” that makes the extended/embedded/enactive mind possible. I will come back to this discussion in the Conclusion (Section 9).

This focus of translation (and interpretation) studies on phenomenology, i.e., “as they are lived”, has been taken up and criticized by Halverson. Halverson (2020) [60] makes out three approaches within CTIS: a computational, a connectionist, and a cognitive approach. She rejects the computational (and connectionist) approach(es) in which cognition is understood as “computational procedures that operate on representational structures in the mind” (p. 38). Rather, Halverson [60] seems to sympathize with the cognitive translatology view in which thinking is performed “by the brain in interaction with the body and the environment” (p. 38). However, Halverson complains that in much research output within the cognitive translatology tradition “the linguistic nature of the task is not in focus at all” (p. 41). She regrets that meaning and communication are in most cases the predominant object of enquiry and requests that “all cognitive translational research programmes must build on a clearly articulated commitment to a view of language and language processing in translation” (p. 38). The current discussion, she says, “has been to emphasize the need for a phenomenological take on translation”. However, she sees no contradiction in calling for a linguistically oriented cognitive translatology while at the same time wishing to situate these processes within a broader, more encompassing world. “[I]t is not impossible” she says, “to model translational cognition in a computational manner and also include both situational parameters and personality-related ones” (p. 41), but “language must be central to our fundamental ontology in all cognitive approaches to translation” (p. 38): “The question is not whether to do so, but how” (p. 49).

This paper develops a perspective on the human translating mind to accomplish precisely this. It explores predictive processing as a possible theory to model enacted and embodied translation processes, with a clear commitment to the linguistic nature of translation while also accounting for the affective nature of human translators. Predictive processing (PP, also known as active inference or predictive coding, Clark [19]) provides a complementary but related view on the mind. While, according to Clark, 4EA cognition emphasizes how cognition emerges from interactions between the body and the environment within a social context, PP focuses on how the brain generates and updates predictions based on sensory inputs. PP thereby acknowledges the importance of the body, the environment, and the (social) context in shaping predictions and perception [19]. Clark, 2023 [19] provides an abundance of evidence that shows how expectations shape our perceptions, our interactions with the environment, and what we take to be true. Similarly, Seth, 2021 [18] maintains that consciousness emerges from the dynamic interaction between the brain, body, and environment.

While PP aligns with embodied and enactive aspects of 4E cognition, it has not been applied within CTIS under this perspective. However, expectations, or anticipatory cognition, have been shown to influence the translator’s decisions and processing strategies on various levels of translation processing: on a sensorimotor level, on a cognitive level, and an affective/phenomenal level of processing [2,28,32]. In this paper, I attempt to integrate those different ideas within a hierarchically embedded architecture of the translating mind.

## 3. Modeling Prediction and Priming in Translation

The modeling of predictions in translation can be addressed on a subjective and on an objective level. It is not clear which level is meant when Schaeffer et al., 2020 [61] (p. 3939) announce the “predictive turn in translation studies”, in which machine learning approaches could be used for modeling human translation processes and for predicting “when and why a translator is having trouble carrying out the [translation] task” (ibid., p. 3940). 

There are two fundamentally different types of models that could predict this “trouble”: An *objective model* elicits and predicts, from an external observer’s (or experimenter’s) point of view, the behavioral correlates of the translator’s trouble. The objective model assumes that an experimenter conducts experiments with one (or more) experimental subjects and evaluates the probability distributions of observed actions or behavioral choices. The resulting model would then allow the experimenter to make informed predictions for similar translation situations. “This is the likelihood of observed behavior given parameters and stimuli—i.e., the likelihood distribution in the objective model” ([16], p. 174).

The *subjective model*, in contrast, would simulate the translators’ ongoing translation processes and (re)produce the causes and effects of the translation trouble, which then result in and can be observed as specific patterns of behavioral data. The “subjective model is assumed to be used by the experimental subject [itself … It] depends on parameters whose value we do not know” ([16], p. 175). However, in PP and AIF, the “general goal is to recover the parameters of the generative model that a subject’s brain uses to produce behavior— the subjective model” ([16], p. 173). 

Fortunately, we can use the objective model to determine how the subjective model produces behavior: we “can invert our objective model on the basis of the behavior we observe to draw inferences about the parameters of the subjective generative model” ([16], p. 173). Based on these assumptions, I outline in Section 4 the components of an objective translation model and map this in Section 5 and Section 6 on a subjective model that is bound to simulate the observed variations of the translation process. 

To date, empirical CTIS has been interested in obtaining objective models of the translation process that describe configurations of parameters indicating how one group of translators compares to another group, or how a different environmental setup impacts translation. Among other things, the focus of interest has been on exploring in what consists translators’ expertise [12,62,63,64], assessing performance variation with different types of texts [65], workspace ergonomics (e.g., [53]), the impact of translation directionality, i.e., L1/L2 translation [66,67], processes of subtitling [68] or spoken translation [69,70], emotional factors [2,58], as well as productivity gains in Machine Translation (MT) post-editing (e.g., [71,72]) and computer-assisted translation (CAT, [55,73]). The aim has been to assess those vital parameters that have an impact on translation effort (e.g., as measured by gaze metrics or keystroke pauses, see below), translation quality, and productivity. 

Expectations and anticipatory processing in translation have been studied under various labels and conditions. Several studies have investigated implicit memory (priming) and explicit expectations in translation. Implicit memory is a mental mechanism that can explain how past experiences influence behavior without the individual being aware of it. It refers to the observation that the retention of information or past experiences can influence behavior, thoughts, or feelings without conscious awareness of the memory. According to Lucas et al., 2019 [74], implicit memory includes cognitive and motor skill learning, habit learning, conditioning, and priming.

Perceptual priming studies stipulate that the physical characteristics of a priming stimulus largely determine the response. Bilingualism studies often investigate how a source language stimulus, a word, sentence, or structure can facilitate effects on successive target language production [75,76,77]. In translation studies, cross-lingual priming effects have been reported on a word and sentence level [78,79,80]. “Shining through” [81,82] can be seen as an effect of perceptual priming. “Shining through” in corpus-based translation studies is a phenomenon where source text (ST) features become apparent in the translated text, of which a translator may, or may not, be consciously aware. I will discuss competing views on this phenomenon in the Conclusion, Section 9. 

Priming effects arise quickly, within milliseconds to seconds or even longer after exposure to a priming stimulus (e.g., a passage of the ST), and depend on various factors, including the strength of the ST-TT association (e.g., how closely the two languages are related or the ambiguity of the translation equivalence), prior experience of the translator, the translator’s emotional state, fatigue, etc. Priming impacts the integration of sensory perception and motor actions, highlighting the intricate connections between perception, action, and cognition. This mechanism thus influences sensorimotor processing, the dynamic interplay between sensory input, motor output, and cognition in translation. 

Another priming effect in translation may be triggered by a translation brief. A translation brief specifies the intended audience and purpose of the translation in the target language. Translation briefs are shown to translators prior to their work and are meant to bias (or prime) translators. A translation brief will activate particular associations that answer to a specific translation expectation. For Nord, 2006 [83] (p. 142), the act of translation depends on the “conclusions the translator draws from the brief … it is no longer the source-text [alone] that guides the translator’s decisions but the overall communicative purpose the target text is supposed to achieve in the target culture”. Also, Sturm, 2017 [84] (p. 16) mentions that “Translation briefs and technical guidelines offer indications both about author intentions and the background of the target audience”. Several authors stress the importance of translation briefs, in particular for MT post-editing, as they have a significant impact on the translation process and product as well [85,86].

However, in contrast to perceptual priming, the translation brief activates associations on a cognitive level. A translation brief can be seen as a type of associative (or conceptual) priming, to the extent the brief specifies terminological preferences or style guidelines, provides background information about the target audience or the intended use of the translated text. This information influences the translator’s interpretation of the ST and directs their decisions with respect to textual constraints, production conditions (e.g., conditions of delivery), and expected translation quality to be achieved in the translation task. A proper interpretation of the translation brief primes, that is, it makes related concepts more easily accessible in memory which influences various cognitive processes such as decision making or problem solving in translation. Thus, Nitzke et al., 2019 [87] highlight the importance of properly understanding the translation brief, and how it is interpreted should be taught during translation education.

In addition, a layer of affect priming may influence emotional responses and the translator’s mood states. Hubscher-Davidson, 2017 [58], for instance, maintains that affect influences judgments in translation. Certain emotions may evoke corresponding emotional experiences that modulate the intensity of emotional responses [2,88].

We thus have three layers of expectation or anticipatory processes in translation: one on a fast sensorimotor level that integrates perception–action loops and perceptual/motor priming, another slightly slower cognitive layer that integrates associative priming of a translation brief with decision making and problem solving, and an affective layer “that makes the world we cocreate with our environments cohere”. 

In the next section, I analyze each of these layers of expectation and anticipation. I show how each layer operates on a different timeline with distinctive pause structures that can be retrieved in the translation process. 

## 4. Pause Analysis and the Task Segment Framework

Human translation production (as other kinds of writing) emerges in terms of typing bursts, that is, sequences of fast keystrokes, that are preceded by a (longer) keystroke pause. Following Muñoz and Apfelthaler [22], in this paper, I refer to the pause between any two successive keystrokes (i.e., successive key-down) as the *Inter-Keystroke Interval* (IKI), which Dhakal et al., 2018 [89] define as “the difference in timestamps between two keypress events”. The duration of an IKI preceding a typing burst has often been assumed to relate to the amount of mental effort required to engage in the successive stretch of typing [21,90]. Whereas smooth typing (i.e., sequences of fast successive keystrokes) indicates unchallenged text (or translation) production, long(er) IKIs fragment the typing flow into segments, indicating translation problems, hurdles, or difficulties.

This assumption is analogous to the eye–mind hypothesis. The eye–mind hypothesis [91] stipulates that the “eyes are where the mind is”, i.e., whatever the eyes fixate on is being processed by the mind. In a similar manner, the pausing-typing assumption suggests that mental processing during a typing break relates (somehow) to the successive typing activities that follow the typing break. Thus, longer IKIs signal higher cognitive effort. 

### 4.1. The Task Segment Framework (TSF)

Several approaches have been proposed to define an IKI threshold, suited to distinguish challenged from unchallenged translation—for a recent overview, see [22,23]. Some authors suggest multiple thresholds that are believed to separate different mental processes [90,92], but it has been controversial what exactly these mental processes are [93]. More often, a single IKI threshold has been assumed to separate two different mental processes: an unproblematic or “default” translation process [45,94] and a challenged or “bumpy” translation mode [95]. 

However, Dragsted, 2005 [64] reports that translators work at different paces and with different typing speeds. She thus suggests using different pause thresholds for different subjects since “comparing all subjects on the basis of the same pause unit value would amount to comparing the motion of a turtle and a leopard as if they both belonged to the same species of animals” ([64], p. 53). Consequently, she proposes a keystroke-pausing threshold (and thus a segmentation method) that depends on the translators’ average production speed, which would reveal certain grammatical structures. She thus calculates the duration of an IKI that segments the keystroke data into sequences of typing bursts in relative times with respect to the individual typing speed.

Muñoz and Apfelthaler, 2022 [22] take up this idea by introducing a *Task Segment Framework* (TSF) that incorporates multiple, translator-relative pause thresholds. They make a distinction between four types of IKI thresholds: Delays, Respites, Task Segment Pauses, and Superpauses (see Table 1). The most basic units of text production, they say, are *motor programs* of 3–4 keystrokes. Motor programs are automatized, embodied routines of fast typing patterns where each IKI is below 200 ms. While motor programs are quick and relatively effortless, Muñoz and Apfelthaler [22] point out that “Motor programs are taken to operate only after typists have settled on the text stretch they want to enter” (p. 10).

Tasks may consist of keystroke sequences inserting new text, changing or deleting existing text, searching for information, etc. (see Section 8 for detailed analysis). Successive Tasks are separated by short breaks which Muñoz and Apfelthaler [22] refer to as *Respites* (henceforth *RSPs*). *RSPs* are accidental, involuntary short stops which do not interrupt the typing flow. On the next level of processing, one or more successive Tasks cluster into Task Segments. Task Segments, in contrast to Tasks, are interrupted by Task Segment Pauses (henceforth *TSPs*) which, according to Muñoz and Apfelthaler, are voluntary, intentional breaks indicating that a translator may need to allocate new resources or address some kind of translation hurdle. *TSPs* thus interrupt stretches of fluent typing. Drawing on (Strömqvist, 1999 [96]), Muñoz and Apfelthaler [22] define the *TSP* as 3 * median between-word IKI and *RSPs* as 2 * median within-word IKI (see Table 1). 

**Figure 1:**
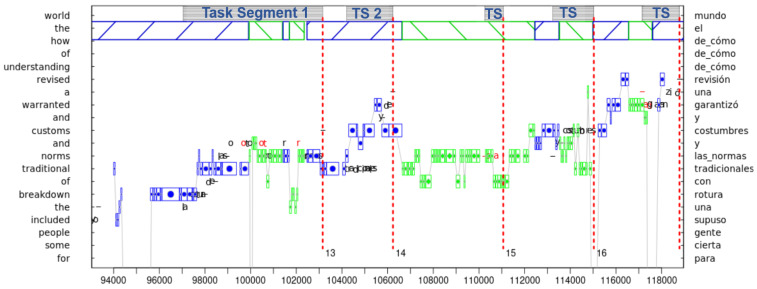
The progression graph shows about 24 seconds (94,000–118,000 ms) of a translation session, visualizing eye movements on the ST (in blue) and eye movements on the TT (in green) as well as keystrokes, insertions (in black), and deletions (in red). The English ST (left Y-axis) and Spanish TT (right Y-axis) are aligned on the word (or phrase) level in the order of the ST (left), from bottom to top. While the English (ST) side shows each individual word in a separate row, the Spanish (TT) side illustrates the phrasal translation alignment. Thus, the two Spanish words “las normas” are here translations of/aligned with English “norms”, while the three English words “understanding of how” are aligned with the two Spanish words “de como”, which are repeated for every ST word in the right Y-axis. The progression graph also visualizes various translation units (TUs) separated by red vertical dashed lines. In the terminology we adopt here from Muñoz and Apfelthaler [22], a TU consists of a typing pause (a Task Segment Pause, *TSP*) followed by a Task Segment (TS). Task Segments are marked as gray bars on top of the graph (the first two TSs are numbered 1 and 2), while the preceding *TSP* is the white stretch between the successive TSs. The graph also visualizes coherent sequences of fixations (fixation units, FUs) on the ST and on the TT, indicated as blue and green striped boxes in the upper part of the figure. While the sequence of FUs indicates how the translator’s eyes switch back and forth between the source and the target windows in the translation editor, TUs elicit the translator’s alternation between pausing and typing. The graph provides an example of how a translator coordinates eye and hand movements in the production of a translation. Note, however, that Muñoz and Apfelthaler do not consider gaze data in their TSF.

**Figure 2:**
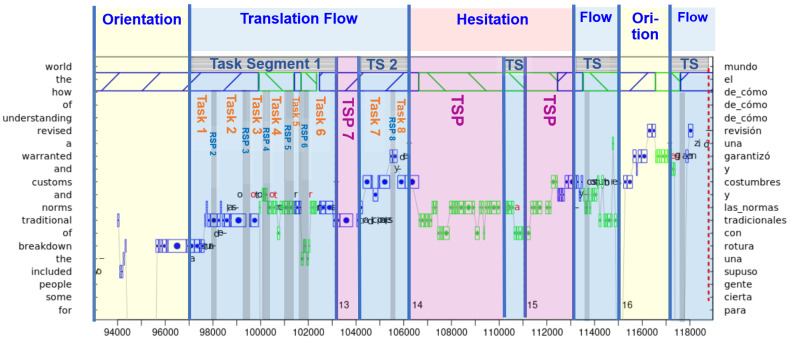
This figure shows a sequence of six phenomenal translation states (Orientation, Flow, Hesitation, Flow, Orientation, Flow), painted on top of the plot of behavioral data from the progression graph shown in Figure 1. Each of the phenomenal states is related to a typical behavioral pattern: A state of Orientation is characterized by forward reading gaze movements on the ST, while Flow and Hesitation states break down into one or more Task Segment(s), separated by a Task Segment Pause (*TSP*). A Task Segment may, in turn, consist of one or more Tasks which are separated by Respites (*RSPs*). This figure shows the embedded nature of translation processes into phenomenal states, Task Segments, and Tasks. It illustrates, among other things, the different shapes of Pauses, Tasks, and Task Segments in the Flow and Hesitation states.

Figure 1 shows a sequence of 24 s of translation process data. An illustration of how the different pauses and typing segments interact is shown in Figure 2. Figure 1 and Figure 2 visualize the process of translating the English phrase “*the breakdown of traditional norms and customs and warranted a revised understanding of*” into Spanish, “*una rotura con las normas y costumbres tradicionales, y garantizó una revisión de cómo*”. The example is taken from the CRITT TPR-DB, Study BML12, translator P06, translating Segment 4 of Test 5 (see also Section 7). It shows the coordination of gaze movements on the source and target text and the typing activities when producing the Spanish translation. The figures illustrate how the translator deals with typing errors in the production of Spanish “*las normas*” in Task Segment 1 and then moving the translation of the pre-nominal English adjective “*traditional*” behind Spanish “*las normas”* in Task Segment 2 (TS 2). The Flow state (see Section 4.2) is followed by a stretch of Hesitation in which the translator apparently scrutinizes cross-linguistic constraints or possibilities, which then leads to inserting “*y costumbres”* (EN: “*and customs”*) before “*tradicionales”* to finally arrive at “*las normas y costumbres tradicionales”*. As noted by one reviewer, the figure also shows that “*garantizó una revisión de como*” may not be the best translation of “*warranted a revised understanding of”.* The figures illustrate the coordination of gazing, pausing and typing behavior, and the realization of different Tasks, Task Segments, and HOF states, as observed in this particular stretch of translation. 

With their TSF, Muñoz and Apfelthaler, 2022 [22] suggest a hierarchical, embedded translation architecture in which two translator-specific pause thresholds, *RSPs* and *TSPs*, depend on the translator’s average typing speed and intentionality. Previous research [21] shows that successive keystrokes are quicker within words (within-word IKIs) than between words (between-word IKIs). Muñoz and Apfelthaler [22] report that IKIs “are also longer at syllable boundaries, and shorter within highly frequent bigrams” (p. 11). That is, average IKIs within words are likely to be shorter than average IKIs between words.

### 4.2. The HOF Taxonomy

While Muñoz and Apfelthaler’s TSF considers only sequences of keystrokes (and their IKIs) for fragmenting the process data into Tasks and Task Segments, a complementary approach for segmenting behavioral translation data has been suggested by Carl et al., 2024 [23] that also assesses gaze data. As we will see, these segmentation approaches unfold on different timelines and account for different levels of mental processes compared to those suggested by Muñoz and Apfelthaler. 

Drawing on the translators’ gaze-hand coordination, as visualized in translation progression graphs (see Figure 1 and Figure 2), Carl et al. [23] develop a novel annotation taxonomy that specifies three basic phenomenal translation states. As Albarracin, 2024 [97] point out, phenomenology is “the descriptive study of the dynamics, structure, and contents of the first-person, conscious experience”. Accordingly, Carl et al.’s HOF taxonomy suggests three basic experiential translation states:

- A state of Orientation is characterized by a long(er) stretch of ST reading, which we take to correspond to [22] Muñoz and Apfelthaler’s Superpause. In a state of Orientation, a translator follows a goal-oriented plan that allows him/her to become aware of possible translation difficulties and to adjust their translation expectations based on the empirically gathered evidence. This may result in more precise priors in successive translation production and higher translation accuracy. The two Orientation states in Figure 2 show short sequences of reading ahead in the ST, which, apparently, were sufficient input for the translator to proceed translation production with a Flow state.
- In a Flow state, a translator engages in largely undisturbed fluent translation. A Flow state may consist of several Task Segments interrupted by *TSPs* that do not exhibit surprise or hesitation. In a Flow state, translation production unfolds with relatively short keystroke pauses and with minimal reading-ahead—thus different from Orientation. A Flow state may be characterized as a type of mindset in which concentration, immersion, and a loss of self-awareness are predominant [98]. Figure 2 depicts three Flow states. The first Flow state consists of two Task Segments, separated by a *TSP* that contains an extended fixation on an ST word currently being translated. Task Segment 1 consists of six Tasks, while the Task Segment (TS 2) is composed of two Tasks.
- A state of Hesitation is triggered in a moment of surprise. It amounts to challenged translation [94] or Pym’s (2017) [95] “bump” mode, which may arise from unforeseen observations that do not match the translator’s expectations. Overcoming a Hesitation may require the adjustment of internal beliefs and/or the modification of translations already generated. A state of Hesitation is, thus, characterized by behavioral patterns that exhibit re-starts, re-reading, or revisions and modifications. The state of Hesitation in Figure 2 shows repeated reading of the same sequence of TT word(s) and a short deletion Task, before the translator comes back to a Flow state.

## 5. Predictive Processing in Translation

While the previous section gives a view from an experimenter’s/observer’s perspective on the translation process, I now draw on predictive processing (PP) to develop a subjective perspective of the translation process from the agent’s point of view. PP maintains that the mind *is* (as opposed to *has*) a model of the world (or environment) which mainly predicts observations and only analyzes them when necessary. Conventional models of the mind “perceive the brain as a relatively passive organ taking inputs from the world and then ‘processing’ them in a predominantly feedforward (outer to inner) fashion” ([19], p. 7). Contrary to this view, PP maintains that “the bulk of what the brain does is learn and maintain a kind of model of body and world—a model that can then be used, moment by moment, to try and predict the sensory signal”. (ibid.: 8) These predictions help structure everything we encounter (see, hear, feel) and create a sense of continuity. We can learn to predict the world, Clark says, “we can learn to do better, until our predictions succeed” (ibid. p. 27). In order to learn or adapt the model when a stimulus does not exactly coincide with a prediction, the agent needs to decide whether the stimulus or the prediction is more trustworthy. In order to do so, the brain constantly estimates a trade-off between these two factors, where the right precision of their balance is of crucial importance. Clark equals this precision with attention. On the one hand, excessive focus on sensory detail may lead to misinterpretations or exaggerations, detracting attentional resources from other tasks or activities. This can have detrimental effects on cognitive functioning. On the other hand, overemphasis on prediction may lead to rigid behavioral patterns and failure to update the internal model that needs to account for changing circumstances. In a similar fashion, consciousness arises, Clark says, from the brain’s predictive processing mechanisms, generating internal models of the world and updating them based on sensory input. In this view, consciousness (conscious experience) can be understood as a form of “controlled hallucination” [18]: the brain generates a predictive model of the world and experiences it as a conscious perception, a principle that has also been referred to as “self-evidencing” [17]. 

In the realm of translation, this perspective suggests that translator’s expertise hinges on the aptness of the translator’s source and the target models. Central to their expertise is the translator’s ability for optimizing and contextualizing the precision function, which reconciles predictions, as, for instance, derived from the translation brief or translation norms, with sensory input from the translation environment, i.e., the source text (ST) itself (see Section 9 for a discussion). Translation expertise is thus contingent upon the translator’s capacity to navigate between the explicit guidelines and expectations of a customer (or the target audience) and the demands of the ST. By effectively synthesizing these components, translators can fine-tune their mental model(s), balancing function and loyalty [99] to the translation by navigating between fidelity to the original ST, the expectations of the target language addressees, and the cultural context.

A PP view on translation would thus underscore the dynamic interplay between predictive processing mechanisms and sensory feedback inherent in the translation process. According to this approach, translators must continuously refine their predictive models based on contextual cues and situational constraints, while also leveraging their perceptual acuity to discern subtle nuances and connotations embedded within the ST. The discussion around Figure 2 suggests that this process concurrently unfolds on three distinct layers of mental description: (1) on the sensorimotor layer, sequences of motor programs integrate into Tasks, (2) on the cognitive layer, Task Segments are planned, processed, and coordinated, and (3) on the phenomenal layer, the agent processes experiential qualities and affective states, integrating sensory perceptions with emotional responses to form a cohesive subjective experience. Analogous to the IKI thresholds in Table 1, Table 2 summarizes some properties of these three processing layers.

## 6. An Embedded Translation Architecture

**Figure 3:**
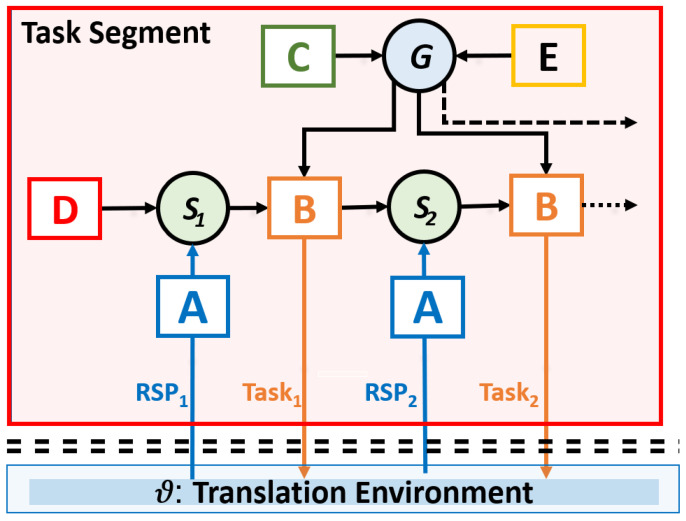
The internal processing structure of a Task Segment on the sensorimotor layer. The Task Segment consists of two successive sensorimotor states *S*_1_ and *S*_2_ (green in black circles). The sensorimotor states *S* are inferring environmental configurations based on sensory input (blue arrows), which are conditioned on the probability distributions of the **A** matrices. Transitions between these states (black arrows) are conditioned on probability distributions specified in a transition matrix **B** (orange boxes) and the execution of a corresponding translation Task (indicated by orange arrows), while the vector **D** encodes priors on these hidden states. The dotted lines indicate the indefinite length of a Task Segment. A Markov Blanket (black double-dashed line) separates the agent’s internal sensorimotor processing layer from outside external states (*ϑ*, see Kirchhoff et al., 2018 [100]). Loops of perception (blue) and action (orange) are presented here as successive events; however, they can also occur concurrently, as, for instance, in Task Segments 1 and 2 in Figure 1 and Figure 2. The sensorimotor layer exhibits a continuous flow of information between epistemic affordances (reading) and pragmatic affordances (typing), showcasing the translator’s dynamic interaction with the environment. AIF conceptualizes predictive processing and anticipatory processing by minimizing the expected free energy of possible future sequences of action that might be generated. An operator, **G,** computes the expected free energy [15,16] for each possible translation continuation within a planning horizon (i.e., *n* steps ahead) and selects the most relevant path (i.e., the next transition in **B**) given preferred observations **C** and habits **E** (see [24]).

In this section, I develop an outline for a subject-based view on the translation process in the form of a translation agent that consists of three layers as discussed in Table 2. The most basic, sensorimotor layer integrates perceptual and motor processes, as shown in Figure 3. On the one hand, sensory input (ST reading) activates lexical representations and primes translation correspondences which are successively typed out ([14,76,77], see also Section 9). On the other hand, motor actions, i.e., TT typing, generate modifications of the translation environment which lead to modified sensory inputs that are monitored and that may change successive predictions, or adjust ongoing translation production. This feedback loop leads to a continuous refinement of the translator’s model in which predictions are tuned to the context and motor action (typing) is adapted according to the perceptual feedback. As depicted in Task Segment 1 in Figure 2, loops of perception and action in translation may be tightly coupled, indicating skilled integration of predictions and guidance of motor actions. 

The activation of lexical translation equivalents may be unconscious, although they can give rise to conscious experiences that are accessible to introspection. For instance, in Task 3 of Task Segment 1 (in Figure 2), a typo seems to have occurred. An attempt to rectify it (deletion of “o”) takes place while the translator is still looking at the ST window (fixations in blue). The eyes then switch to the TT window (fixations in green), and in the successive Tasks 4, 5, and 6, several follow-up typos (insertion and deletion of “ot” and “r”) are produced and addressed. The translator seems to have noticed the first typo in Task 3 without visual feedback, probably due to the sensing of an incorrect execution of a motor program. Successively, the translator switches visual attention to the TT and monitors the typing activity, which indicates the tight integration of sensorimotor processes. 

Another pattern of sensorimotor integration can be observed in Task Segment 2. Here, the eyes read a few words ahead in the ST while the hands still type out a stretch of legacy TT. Also this Task shows a tight integration of perception–action loops, which allows for seamless translation production: while the translator takes in a new piece of ST, the hands still type out a piece of TT from the previous chunk. Jakobsen, 2005 [101] reports that such patterns of “peak performance” can be observed over stretches of more than 100 keystrokes for experienced translators.

The anticipation of optimal production processes is modeled within AIF through minimizing chances of suboptimal state transitions. These state transitions, from S_i_ to S_i+1_ in Figure 3, are specified in matrix **B**. The operator **G** in Figure 3 takes as input a definition of preferred behavior (as specified by vector **C**) and a definition of habits (e.g., long context planner/head starter/online revision, specified by **E**). Given these constraints and the current configuration of the translation model, the **G** operator aims at producing an optimal path though the state space by adapting the transition probabilities in **B**. At the sensorimotor level, this may result in the realization of different Task trajectories, as exemplified in Figure 1 and Figure 2.

**Figure 4:**
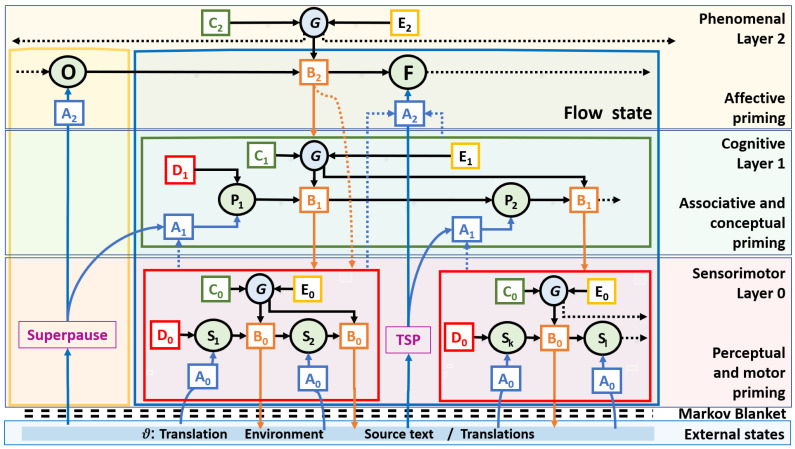
This figure depicts the interaction between the three internal layers of the translation agent and the external states in the environment (*ϑ*). It shows a possible instantiation of two successive phenomenal states, Orientation (**O**, big yellow box) followed by Flow state (**F,** big blue box). Three internal processing strata unfold concurrently on different timelines, on sensorimotor, cognitive, and phenomenal layers. Each layer consists of a sequence of states (green circles with black lines) and transitions between them (**B** matrices in orange boxes). Transitions between successive states are conditioned on actions, indicated by orange downwards arrows. Actions condition resources in the downstream lower layers. The dotted downward orange arrow indicates action(s) by which the phenomenal layer may also directly impact sensorimotor processes. In one possible instantiation of the architecture, each state infers the status of the translation environment based on sensory input (blue solid arrows), conditioned on the current internal state(s), as specified by the blue **A** matrices [102]. Alternatively, each processing layer may (also) obtain feedback information from the embedded layer(s), as indicated by the blue dotted upwards arrows. As suggested in the AIF/PP literature, the trade-off between the internal predictions and the sensory input may be balanced by a precision value.

Figure 4 depicts the hierarchical architecture of a translation agent with three embedded processing layers. Figure 4 shows three interconnected internal processing layers, an external state that consists of the translation environment, the source and target texts, and a Markov Blanket that separates the external and the internal states (Kirchhoff et al., 2018 [100]). The three internal layers have similar structures as discussed in the context of the sensorimotor layer. Each layer specifies a sequence of internal states and transitions between them. Each layer also implements an AIF mechanism capable of computing an optimized path through the state space, given layer-specific preferences and habits. 

The cognitive layer involves higher-order processes including memory, reasoning, problem solving, or the generation of complex thoughts The states *P* on the cognitive layer in Figure 4 activate the respective executive functions such as planning, decision making, problem solving, cognitive control, self-regulation, etc. These functions may also imply metacognition, including the need for monitoring, or the regulation of cognitive processes. These cognitive states may play a crucial role in goal-directed behavior by selecting/activating appropriate subsections of the translator’s model that are attended to on the sensorimotor layer. The cognitive and the sensorimotor layers may thus dynamically interact in support of adaptive behavior and cognition – see Section 9 for a discussion. 

The orange arrows in Figure 4 that emanate from the **B_1_** matrices may activate or control resources on the sensorimotor layer, while the blue dashed lines provide feedback from the sensorimotor layer to the cognitive layer. In this architecture, the sensorimotor layer interacts with the environment, while the cognitive layer builds upon this foundation to support more complex processes and behaviors. 

The third, phenomenal layer encompasses subjective conscious experiences. PP and 4EA theories emphasize the close connection between sensorimotor and phenomenal processes to play a foundational role in shaping conscious experience. On the one hand, sensorimotor and cognitive processes can influence conscious awareness and emotional states. These connections are indicated as blue dotted arrows in Figure 4. That is, sensorimotor attention (i.e., sensorimotor precision) may influence how sensory stimuli are experienced and interpreted, while cognitive processes such as conceptualization, memory or thought may structure conscious experiences and phenomenal awareness. On the other hand, phenomenal experience can also influence cognitive and sensorimotor processing, as indicated in orange arrows in Figure 4. For instance, emotional states can impact memory and decision making, biasing cognitive and/or sensorimotor processing toward emotionally salient stimuli (indicated by the orange dotted lines). Phenomenal states can influence action selection, bias perceptual processing, or alter the interpretation and integration of sensory information so as to align with subjective experiences. They can contribute to the integration and coupling of sensory and motor processes, creating a dynamic feedback loop between perception and action.

## 7. Pausing Structure across Languages and Translators 

In this and the next section, I illustrate properties of the pausing and keystroke data structure that the proposed translation agent might be expected to produce. I extract a subset of 54 English-to-Spanish and English-to-Arabic translation sessions from the CRITT TPR-DB, compute *RSPs* and *TSPs*, and provide summary information of properties of Tasks and Task Segments. The data show interesting similarities and differences across translators and languages in the realization of *RSPs*, *TSPs*, Tasks, Task Segments, and HOF states. 

In this section, I illustrate the IKI structure of the logging data and illustrate cross-lingual and cross-personal differences. In the next Section 8, I discuss the properties of the state spaces on the three layers of the embedded architecture.

### 7.1. The Empirical Data 

The CRITT Translation Process Research Database (CRITT TPR-DB, [31]) is a publicly available repository of translation process data that are freely available under a Creative Commons License (CC BY-NC-SA). It is hosted at sourceforge.net with extensive documentation on the CRITT webpage (https://sites.google.com/site/centretranslationinnovation/home, accessed 12 July 2024). The TPR-DB contains currently more than 5000 translation sessions: more than 600 h of text production with recorded keystroke logging, mostly written translation sessions but also authoring and spoken translation (sight translation, reading aloud, etc.). Many studies also have gaze data. 

In this study, I investigate keystroke data of from-scratch translation sessions from the MultiLing sub-corpus. The MultiLing corpus (https://sites.google.com/site/centretranslationinnovation/tpr-db/public-studies#h.p_iVVuCQOHJx2O, accessed 12 July 2024) consists of six short English source texts (STs), each between 110 and 160 words. The six STs have 847 words in total and are translated into several languages under different translation conditions. Table 3 compares the IKIs of from-scratch translation sessions from the six MultiLing texts into five languages (English to Arabic, Spanish, Danish, Hindi, German). Table 3 shows the TPR-DB study names with their target language, the number of total keystrokes in each study, the total duration in hours, the number of sessions and different translators, as well as the mean and median lag of time between successive keystrokes (IKIs) in ms. It shows that Danish translators (KTHJ08) are (on average) the fastest typists in our dataset: a total of 24 Danish translators needed a total of 7.7 h to produce 69 translations with 72.383 keystrokes (383 ms per keystroke on average). Hindi translators (NJ12), in contrast, were the slowest in this dataset; they needed, on average, 1223 ms per keystroke, more than 3 times longer. 

**Figure 5:**
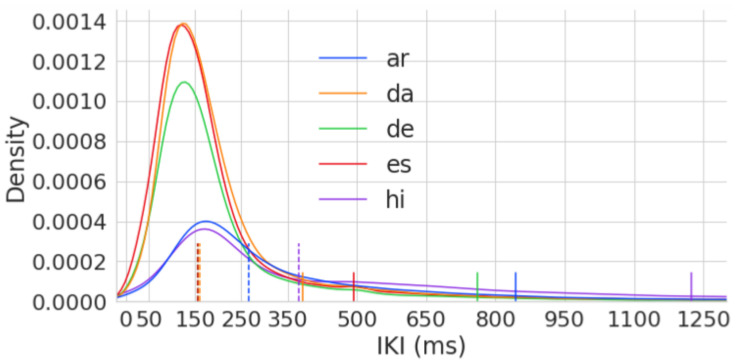
Density of IKIs for five languages.

Figure 5 shows the distribution of the IKIs of these five languages from Table 3. All language pairs show a somewhat similar IKI distribution, with one peak and a very long tail, heavily skewed towards the right. Two language pairs, English-to-Spanish (es) and English-to-Danish (da), have peaks (Modus) around 140 ms, while English-to-Arabic (ar) and English-to-Hindi (hi) have a much flatter distribution with one IKI peak around 160 ms. The mean and median IKIs are shown in Table 3 and marked in Figure 5 in solid lines and dotted lines, respectively.

Given that the Spanish and Arabic datasets show quite different IKI distributions, I only look at those two target languages in the reminder of this paper. The English-to-Arabic translations were collected from 22 experienced PhD students at Kent State University. The English-to-Spanish data were collected in 2012 from translation students and have been used since then in numerous studies ([40,103,104], and others). We only use the from-scratch translation data in this study.

### 7.2. Word Boundaries, RSPs, and TSPs

Every recorded keystroke in the CRITT TPR-DB is assigned a key-down timestamp [89], where an IKI is the lag of time between two successive key-down events. Additional information is computed for each keystroke, such as the index of the segment in which a keystroke was produced, the TL word that it produced, the corresponding translation equivalent in the ST, etc. This information is, however, not used in this study. 

For the sake of computing *RSPs* and *TSPs*, a *word-boundary* keystroke is defined to be any of the following keystrokes (blank spaces are mapped on the underscore “_”):

` “’_.!?:=@$%&*()[]{}

A keystroke is considered *within-word* if it is not a word-boundary keystroke and neither preceded nor followed by a word-boundary keystroke. A *word initial* keystroke is the first keystroke of a word (i.e., not a word-boundary keystroke), and a *segment initial* keystroke is the first non-word-boundary keystroke of a new segment. Note that a first-segment keystroke is usually also a word initial keystroke. 

A *within-word pause* (*WP*) is an IKI preceding a within-word keystroke. The IKI preceding a word initial keystroke is defined to be the *between-word pause* (*BP*), and the IKI preceding a segment initial keystroke is the *between-segment pause* (*SP*). 

As translators have different typing skills and typing styles, Muñoz and Apfelthaler [22] expect different *WPs* and *BPs* for every translator. In addition, it has often been assumed [21,22] that *WPs* are shorter than *BPs* and *BPs* are shorter than *SPs.* Following the discussion in Table 1, values for *RSPs* and *TSPs* are computed for each translator *i* separately, based on their median $WP_{i}$ and the median $BP_{i}$. Thus, as previously discussed, Muñoz and Apfelthaler define ${RSP}_{i}$ and ${TSP}_{i}$ as follows:

$${RSP}_{i}=2*median(WP_{i})$$

$${TSP}_{i}=3*median(BP_{i})$$

Table 4 shows several values (min, max, mean, median) for *RSPs* and *TSPs* for the 22 Arabic (ar) and the 32 Spanish (es) translators. The Table shows that the values for the Arabic translators are much higher (almost twice) than for their Spanish colleagues.

The minimum *RSP* duration is 220 ms for Spanish translator P11 in our data, which is just above the assumed value for a Delay (200 ms, see Table 1). The maximum *RSP* duration is 1032 ms (for translator P18 in the Arabic data). The minimum *TSP* duration is 423 ms and the maximum is 2388 ms, which is, by coincidence, achieved by the same two translators. 

### 7.3. Relating RSPs and TSPs

**Figure 6:**
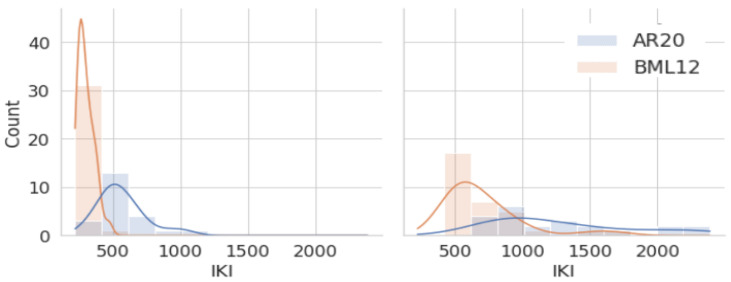
Distribution of *RSPs* (**left**) and *TSPs* (**right**) for Spanish (BML12) and Arabic (AR20) translators.

Figure 6 shows the distribution of *RSPs* on the left and *TSPs* on the right for 32 Spanish and 22 Arabic translators. It shows that Arabic translations have much larger variability, i.e., a flatter distribution, than Spanish. As Muñoz and Apfelthaler assumed, the durations of *RSPs* are—for every translator—shorter than those of *TSPs*. 

**Figure 7:**
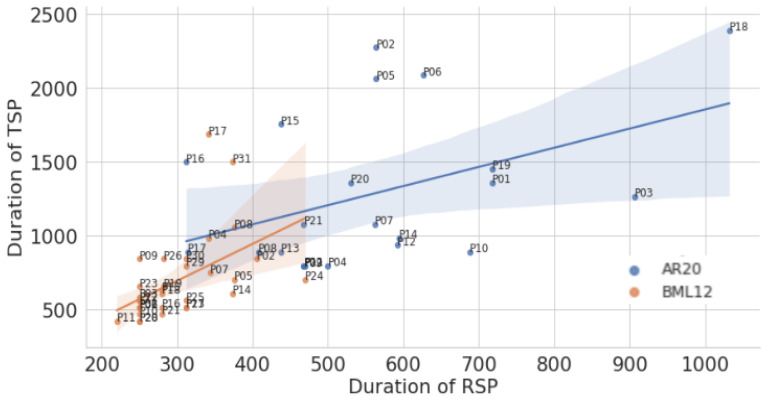
Correlation of RSPs and TSPs.

Figure 7 shows that *RSPs* tend to correlate with *TSPs*. This correlation is significant for Spanish (Spearman τ: 0.68, *p* < 0.0001), while it is not significant for Arabic (Spearman τ: 0.40, *p*: 0.065). 

**Figure 8:**
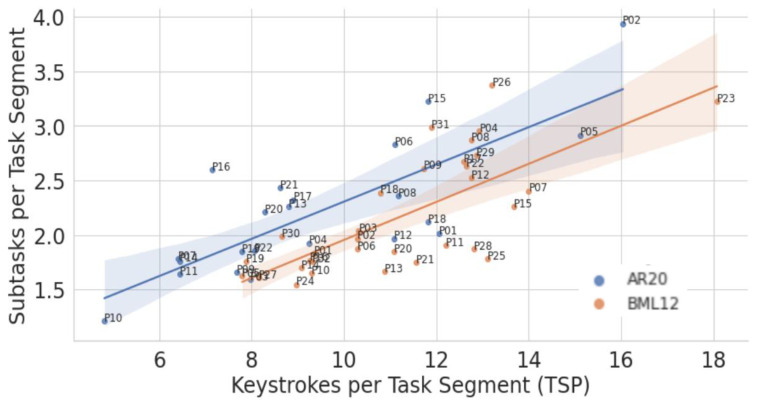
Correlation of total number of keystrokes per Task Segment and number of Tasks.

As plotted in Figure 8, there is a strong correlation between the number of Tasks within a Task Segment and the number of keystrokes produced in that Task Segment (τ: 0.74 and τ: 0.73, *p*: 0.000 for Arabic and Spanish, respectively). While, on average, Arabic and Spanish translators engage in 2.2 Tasks per Task Segment, Arabic translators show, also here, a larger variation (min 1.2 and max 3.9, median 2.1) than Spanish translators (min 2.1 and max 3.4, median 1.94). 

Spanish translators also produce, on average, more keystrokes per Task Segment than Arabic translators do. A Spanish Task Segment contains between 8 and 18 keystrokes (mean 11.2), while an Arabic Task Segment has between 5 and 16 keystrokes (mean 9.4). However, while the mean number of Tasks is almost identical for Arabic and Spanish Task Segments, (i.e., 2.2), a Spanish Task has between 3.9 and 7.4 keystrokes (mean 5.3), while an Arabic Task has between 2.7 and 6.0 keystrokes (mean 4.3), per translator. 

### 7.4. Personal Pausing Profiles

As Figure 7 and Figure 8 indicate, the keystroke distributions seem to discriminate not only between the two language pairs, but also between every individual translator. A two-sample Kolmogorov Smirnov Test (KS2) shows that in 78% of the cases, different translations from the same translator were indeed (correctly) recognized as samples from the same population (i.e., the same translator), while in 96% of the cases translations from different translators were correctly recognized as samples from different populations (i.e., different translators). It is thus interesting to note not only that there seem to be differences between languages but also that each translator seems to have their personal pausing structure. However, at this stage more research is required to determine what exactly these different personal pausing structures represent.

We also observe a slight negative effect of Task Segment length on the number of keystrokes produced per Task: as the number of Tasks per Task Segment increases, so decreases the number of keystrokes per Task. This effect is significant for Spanish (τ: −0.52, *p*: 0.002) but not for Arabic (τ: −0.18, *p*: 0.41), which may have to do with the larger variability in the data and the smaller number of observations for our Arabic data set. 

## 8. Task Structure across Processing Layers

As discussed in the context of Figure 2, each Task Segment is a sequence of Tasks where a Task is separated by an *RSP*. Following our architecture in Figure 4, sequences of Tasks are realized on the sensorimotor layer, while the planning and execution of Task Segments pertain to the cognitive layer. Inspired by Muñoz and Apfelthaler’s Task typology, in this section I first assess a taxonomy of Tasks and Task Segments and then look at their relation to the HOF states. 

### 8.1. Types of Tasks and Task Segments

Muñoz and Apfelthaler distinguish between several types of Tasks that involve different types of keystrokes. They suggest the following types of Tasks (subtasks): ADD (adding new text), CHANGE (changing the text), SEARCH (searching for information), HCI (human–computer interaction). However, this list seems to be open to further extension and/or more fine-grained differentiation. In this study I adopt the following three Task types: an insertion Task, **A**, has only insertion keystrokes (corresponds to Muñoz and Apfelthaler’s ADD), a deletion Task, **D,** has only deletions (not considered by Muñoz and Apfelthaler), and a change Task, **C**, has insertions and deletions (corresponds to [22] Muñoz and Apfelthaler’s CHANGE). I omit the SEARCH Task since the setup of our translation sessions did not allow for external research. 

**Figure 9:**
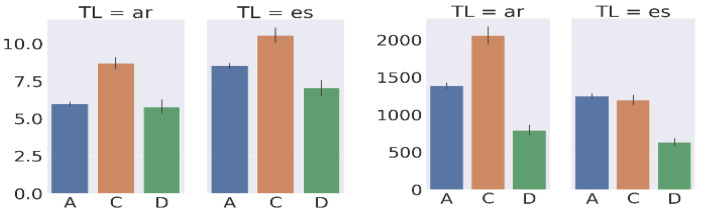
The number of keystrokes (**left**) and durations (**right**) for the three types of Arabic and Spanish Tasks, **A**, **C**, **D**. Our data show more keystrokes and shorter timespans for the Spanish Tasks.

Figure 9 shows the average duration and number of keystrokes of the three types of Tasks for the Spanish and Arabic translations. The figure shows that there are systematic differences between the Spanish and the Arabic Tasks: on average, all types of Tasks **A**, **D**, and **C** have more keystrokes for Spanish as compared to Arabic translation, but the average duration is longer for Arabic than for Spanish. This observation corroborates findings from the previous section, which showed that Spanish translators produce their translations more quickly than Arabic ones. 

Task Segments consist of sequences of Tasks. As each Task has a label—in the current setting, **A**, **D,** or **C**—I take the concatenation of Task labels that are realized within one Task Segment to label (i.e., characterize) the Task Segment. There are altogether 10,356 Task Segments in the joint Arabic and Spanish translation data with 892 different Task Segment labels. The mean and median durations of a Task Segment are 6777 ms and 5781 ms, and the median, mean, and maximum numbers of Tasks are 11, 14, and 61 per Task Segment, respectively. 

More than 93% of these Task Segment labels—that is, 833 different labels—occur less than 10 times (that is, each Task Segment label covers < 0.1% of the data). Together, they account for 13.8% of the data, or 1426 Task Segments. For the 20 most frequent types of Task Segments the mean and median durations are 3479 s and 3183 s, respectively, and the number of Tasks is, on average, 3.0. Thus, just as the pause structure (Figure 5), the distribution of the Task Segment labels also has a very long tail. Table 5 gives a summary of the 11 most frequent Task Segment labels which make up 75% and 71% percent of Spanish and Arabic data, respectively. 

Table 5 provides a summary for the most frequent Task Segment labels (composed of Task labels **A**,**D**,**C**). It shows the total number of occurrences per Task Segment label, the percentage of Spanish and Arabic data, as well as the duration (in ms) of the Task Segment (DurTS), the average IKI, and average number of keystrokes per Task (KeyT, the average keystrokes per TS, would hence be the product of number of Tasks * KeyT).

There is a very strong correlation between the proportion of the Spanish (%es) and the Arabic (%ar) Task Segment labels (r = 0.998), which may indicate that these labels are language- and translator-independent. Thus, the most frequent Task Segment, 38% and 36% of the Spanish and Arabic data, respectively, consists of a single ADD Task **A**. Table 5 indicates that there are, on average, 5.33 keystrokes for this Task with an average IKI of 173 ms. However, given the discussion in the previous section, there may be interaction effects between the average duration, the two languages and the individual translators, a relation that should be investigated in more detail in future research.

As previously mentioned, the average number of keystrokes per Task decreases as the length of Task Segment increases. While there are, on average, 5.33 keystrokes per Task for a TS with a single **A** Task, there are 5.13 keystrokes per Task if the Task Segment consists of two **A** Tasks (**AA**), 4.81 keystrokes if the Task Segment has three **A** Tasks (**AAA**), 4.67 for four Tasks, etc. On the other hand, the IKIs tend to increase as the Task Segments become longer, which suggests that typing slows down or becomes more interrupted as the Task Segment is composed of more Tasks. 

Olalla-Soler, 2023 [105] assumes that Task Segments that consist only of a few ADD Tasks constitute *default translations* [45]. He observes that 67.8% of his Task Segments were default translations which contained 69.5% of the words. The observations in our data show that slightly more than 60% of the Task Segments are **A**-only, and they cover around 44% of all the keystrokes. 

It is also interesting to note that IKI profiles—that is, the keystroke pausing structure, as shown, e.g., in Section 7, Figure 7 and Figure 8—seem to be typical for specific languages and translators. Our preliminary analysis of Task Segment labels, in contrast, suggest that the frequency and order of Task types that are realized within a Task Segment seem to be language- and translator-independent. 

### 8.2. HOF States, Tasks, and Task Segments

In Section 4 and Section 6 it was assumed that each layer in the agent’s hierarchical architecture operates on a different time scale and thus with different pausing structure and duration. The sensorimotor layer organizes sequences of Tasks, while the cognitive layer organizes sequences of Task Segments. As discussed in Section 4, on the phenomenal layer, the architecture specifies three broad experiential translation states. 

In previous work [23], we have manually annotated data from eight Spanish and six Arabic translation sessions with HOF state labels. The annotation process is described in detail in [23,106]; here, I analyze the annotations in light of the architecture in Figure 4, without giving further justifications or explanations of the HOF taxonomy. 

Table 6 provides an overview of the annotated states in the eight Spanish and six Arabic translation sessions. Despite the different absolute numbers of states in the two languages (a total of 606 and 292 state annotations, respectively), it is interesting to note that the percentages of H, O, and F states are almost identical in the two languages, as indicated in the % columns in Table 6.

Table 7 shows the transition matrix between two successive HOF states, in percentage, for Arabic and Spanish data on the left and right, respectively. The rows indicate the state at time *i* (from where the transition starts), while the columns indicate the transition probability into the next state at time *i + 1*. As can be seen, the most likely pattern, according to this table, is a loop over the Orientation (O) and Flow (F) states (marked in bold). Only in 16% and 14% of the cases for Arabic (ar) and Spanish (es), respectively, is an Orientation state followed by a Hesitation. Here, too, the transition patterns in both transition matrices for Arabic and Spanish are quite similar, with the only exception, perhaps, that Arabic translators transition more often from a Hesitation to a successive Orientation (21%), while this is much less likely for Spanish translators (9% of the cases). However, in both cases, perhaps not surprisingly, the highest chances are that a translator will try to arrive at a Flow state (F).

Table 8 shows the distribution of **A**, **D**, and **C** Tasks in the Hesitation (H) and Flow (F) states for the two languages. According to this table, as can be expected, the Flow states are clearly dominated by **A** Tasks (>80%), while they are more equally distributed (54% and 53%) as compared to the sum of deletions and changes during Hesitation.

Table 9 shows the six most frequent Task Segment labels for F and H states in the two languages. These 2*six types of TSs for the different HOF states account for roughly 75% of the data. As for the correlation reported in Table 5, this table also shows a very strong correlation between the Task Segment labels for the Arabic and Spanish Flow states and—slightly less—for the Arabic and Spanish Hesitation states. Thus, the correlation coefficient for the first 20 TS labels in Arabic and Spanish Flow states amounts to r = 0.993, while it is still r = 0.968 for the Arabic and Spanish Hesitation states (not all labels shown in Table 9). The correlation between Flow and Hesitation states is still strong but lower, r = 0.76. 

This seems to indicate, as before, that Tasks and Task Segments realized in HOF states across different languages seem to depend on the Task itself, rather than on the language or (presumably) the translator. 

### 8.3. Time Structure of HOF States

While the frequency and distribution of Task and Task Segment labels do not seem to depend on the target language, in this subsection, I assess whether there are significant differences in the translation processes that underly the realization of HOF states in Arabic and Spanish. Table 10 and Table 11 provide values for the duration as well as the number of keystrokes, Tasks, and Task Segments of Flow and Hesitation, respectively. The tables indicate the mean, minimum, and maximum values of these variables.

As Table 10 shows, the duration of Arabic Flow states is approximately twice as long as the Spanish ones (Dur/F). There are more keystrokes per Arabic Flow state (Key/F), more Task Segments (TS/F) and Tasks (T/F) in the Arabic data, and the variation of these parameters is higher for the Arabic as compared with the Spanish data. However, the number of keystrokes per Task (Key/T) and the number of keystrokes per Task Segment (Key/TS) seem to be lower for Arabic than for Spanish. As in the previous discussion, this indicates that Arabic translators are slower, and their production proceeds with more disruption as compared to the Spanish translators.

A slightly different pattern can be observed for Hesitations, as shown in Table 11. As for Flow states, the duration of Hesitations is also longer for Arabic than for Spanish, and there are more keystrokes and Task Segments in Arabic than in Spanish Hesitations. However, the number of keystrokes per Hesitation (Key/H) in the Arabic data is approximately ½ as compared to Flow states (17.68 vs. 36.58), while for the Spanish data this ratio approaches 1/9 (3.19 vs. 29.24). This large difference may indicate a less clear discrimination between states of Hesitation and Flow in the Arabic data, an observation that might merit more investigation in future research. 

The potentially blurred boundary between H and F states in the Arabic data is also corroborated by the fact that Spanish translators spend approximately 18% of their time in Flow states in *TSPs,* while the *TSP* time in Hesitations is 3 times higher, 54%. That is, Spanish translators spend approximately 54% of their Hesitations time in (presumably) reflection or visual search (i.e., no keystrokes produced) and accordingly 46% in the completion of Task Segments (i.e., typing/modification). Arabic translators, in contrast, spend 26% of their time in the Flow state in pausing (i.e., *TSPs)*, while this is the case only for 45% during Hesitation. The boundary between reflection and typing in Flow and Hesitation seems to be less pronounced in the Arabic data.

Finally, the slower (or more meticulous) pace of Arabic translators is also obvious in the duration of Orientation states. As shown in Table 12, the durations of Orientation states observed in the Arabic data amounts to approximately twice that of their Spanish colleagues.

Thus, while the temporal structures of Tasks, Task Segments, and HOF states show clear differences between languages and (potentially) translators, this does not seem to be the case for the aggregate Task and Task Segment labels. However, more in-depth research is required to corroborate these results and to develop more fine-grained classification taxonomies that may elicit the hidden relations in more detail. 

## 9. Discussion and Conclusions

This paper presents a hierarchically embedded model of human translation processes that aligns with the principles of active inference (AIF) and predictive processing (PP). The central idea of PP/AIF is that the mind constructs a model of the environment to guide and evaluate behavior. PP and AIF posit that the brain is a prediction machine (Friston et al., 2017 [15], Seth, 2021 [18], Clark, 2023 [19]) that continually generates and updates predictions about incoming sensory information. The incoming information is offset against prior knowledge (or expectations) so as to minimize prediction error. PP thereby acknowledges the role of the body and the environment in generating and testing predictions. That is, PP endorses that cognition emerges from the interaction between the organism and its environment. 

This paper suggests a formal model that allows us to specify and implement these assumed translation processes from a subjective perspective, in the form of a translation agent.

### 9.1. The Subjective View

More than 30 years ago, Neubert and Shreve, 1992 [7] proposed a multi-layer model of human translation processes which, they suggest, consists of (1) unconscious mental operations that “invoke pre-existent internalized courses-of-action” (ibid. 52) and (2) translation strategies and procedures that, according to them, constitute “generalizations about typical courses-of-action”, assisted by “information recall, storage, and integration procedures, and monitored by pattern-matching and planning procedures” (ibid.). They maintain the following:

*“We must understand how the mental processes act upon and influence one another as they are applied. It is clear that these procedures are not part of a simple sequential process; they are not activated in a fixed order. Their temporal and sequential relationships are more complex. The entire convoluted process resembles a network more than a chain”*

(p. 50)

The suggested PP/AIF agent advances this project in a formalized manner. The model of the proposed translation agent is described in Figure 4. It consists of three embedded layers: a sensorimotor layer, a cognitive layer, and a phenomenal layer. Each of the phenomenal HOF states in Figure 4 is associated with embedded cognitive and sensorimotor processes with characteristic properties of the current phenomenal translation state. The cognitive layer simulates cognitive functions, which include memory, attention, and decision making. The sensorimotor layer simulates the integration of sensory modalities (reading) and motor actions (typing). As discussed below, different modes of integration of these three layers allow us to simulate the coordination of perception, action, cognition, and phenomenal experience in translation. 

The hierarchical architecture and its three embedded processing layers in Figure 4 are depicted as Partially Observable Markov Decision Processes (POMDPs, [24]). Within their introduction to POMDP, Heins (2022, https://github.com/infer-actively/pymdp/blob/master/docs/notebooks/active_inference_from_scratch.ipynb, accessed on 12 July 2024) operationalizes the interaction between the organism and its environment in the following way:

1. Sample an observation from the current state of the environment;
2. Perform inference over hidden states through free-energy minimization;
3. Calculate the expected free energy of actions *G*;
4. Sample action from the posterior over actions;
5. Use the sampled action to perturb the generative process and go back to step 1.

In the translation context, this cycle suggests that a translator comes into a translation situation with some prior knowledge of the translation assignment and a set of preferences and expectations that lead to a course of action. Translators may update their internal models based on their observations (perceptions) and/or they may adapt their (preferred) course of action according to the requirements/constraints of the translation environment, which includes the source and target texts.

Each layer in Figure 4 consists of a network of states (in circles) and transitions between them (**B** matrices). Transitions between successive states are conditioned on actions (orange arrows). The states and transitions of the embedded layers may be initialized by higher states. Da Costa and Sandved-Smith, 2024 [102] suggest that higher states can infer the status of lower (embedded) states by assessing changes in the environment via sensory input, as indicated through blue solid lines and conditioned by the **A** matrices. This leads to the conclusion that what an agent perceives is (partly) due to how it acts on the environment and hence through “action that gives an organism a grip on its environmental affordances” ([107], p. 52). Alternatively, the “simulation theory of conscious experience” (ibid. 47 ff) suggests an architecture in which internal feedback loops inform the higher layers about the embedded layer’s internal states. These feedback loops are indicated in dashed blue lines in Figure 4. They transfer information from the lower level, independent from configurations of the environment, and are thus prone to produce “inferred fantasies” [107] or “controlled hallucinations” [18] or, in a slightly more positive view, simulate “metacognition” [102]. It may also be possible that both types of top-down and bottom-up connections concurrently exist and complement each other, depending on the concrete implementation and the value of a precision parameter. 

The agent proposed in this paper aims at simulating observed variation in translation behavior, as, for instance, outlined in Section 7 and Section 8. It has the potential to shed new light on the mechanisms underlying human translation performance. It might allow us to generate novel hypotheses and explanations with respect to various questions in CTIS and to empirically test and validate them as a computational simulation of the AIF agent. 

For instance, the PP view may suggest that translation expertise, a topic of constant interest among CTIS researchers [108], depends on how well a translator has acquired a model of the ST with its translation possibilities into the target language [109,110] and how skillful a translator can optimize and contextualize the precision function to balance the attention between their own predictions and the sensory input from the translation environment. The PP agent may be suited to simulate performance variation in L1/L2 translation (directionality), taking into account characteristics of the language pair as, e.g., discussed in Section 7 and Section 8. It might be suited to simulate and assess the impact of working memory or emotional and cognitive ergonomic factors such as MT post-editing, CAT usage, etc. Some of these issues are discussed as follows.

### 9.2. Shared Representations and Lexical Co-Activation

One long-standing topic under discussion within CTIS aims at explaining the observation that some translations seem to be easier and faster to produce than others. Ease of translation is related to the automatization of translation patterns, a phenomenon that has been reported for a long time by many translation scholars. Two competing hypotheses have recently been suggested to explain this phenomenon. The first hypothesis (hypothesis 1) explains fluent/automatized translation production through cross-lingual structural similarities of the source and target that lead to subconscious priming of “shared representations” [14,80,111]. The activation of shared representations makes it successively easier for the translator to enter a state of fluent translation production. This hypothesis rests on the assumption of shared cross-lingual syntactic similarities and a mechanism of semantic/conceptual priming at the level of abstract syntactic structures and grammatical patterns that facilitate translation production.

The second hypothesis (hypothesis 2) suggests that specific translation patterns are learned and automatized through repeated practice. These patterns consist of “easily accessible routinized knowledge” ([45], p. 190) that can (but must not) consist of cross-lingual syntactic similarities. This hypothesis relies more on the sensory and perceptual features of the stimuli rather than their cross-lingual semantic or syntactic similarities.

With the three-layer architecture of the proposed translation agent, the underlying assumed processes of these two hypotheses may be conceptualized quite differently. The first hypothesis draws on resources that are most likely located at the cognitive layer, whereas the second hypothesis relies more heavily on automatized sensorimotor processes and perceptual priming. These hypotheses may boil down to different ways of coordinating and integrating the sensorimotor and the cognitive layers and to conceptualize the choices made within those layers, which may be implemented and verified in the agent. However, while these hypotheses predict different modes of priming, AIF goes even a step further by positing that an agent can actively, consciously or non-consciously, choose the priming input - for instance a piece of the ST - which reduces its surprise (i.e., the free energy). That is, AIF stipulates that an agent (e.g., a translator) will select a visual stimulus which allows her to proceed most effectively in subsequent (translational) action, and she will engage in action that reduce surprise of the successive observation, a perception-action loop referred to as self-evidencing (see below). 

A related question concerns the origin of “shining through” effects ([81,82], see Section 3), in which the grammatical structure of a source sentence may leave traces in the translated TT. The first hypothesis that explains this observation is similar to the first one in the previous paragraphs, assuming that “shared representations” prime translation production so that the translator will be biased to produce translations that are structurally similar to the ST, if the TT allows for such structures. The second hypothesis suggests that word-for-word translation (i.e., serial lexical co-activation) facilitates the translation process, without necessary recourse to cross-lingual structural similarities [112]. In both cases, a shining through effect will be visible in the translation, but in the second case, the similarity of syntactic structures is merely a side effect of word-for-word translation, rather than a (compulsory) starting point of translation. 

Here, too, the assumed processes may be implemented differently in the proposed agent’s architecture, putting the burden on the cognitive layer (first hypothesis) or on the sensorimotor layer (second hypothesis). Differences might result, for instance, from assumptions about the prediction horizon on the sensorimotor layer and its access to entrenched sequences of, e.g., n-gram translation patterns, or from assumptions about the prediction horizon on the cognitive layer that facilitate/monitor the compliance of the translation outcome with respect to the structural requirements and the anticipation of conceptual language constraints.

The artificial translation agent, constrained to comply with basic properties of the translation process, as, e.g., described in Section 7 and Section 8, might shed new light on this sensorimotor/cognitive controversy. 

### 9.3. Hesitation and Default Translations

Not unlike the three-layer architecture of the proposed translation agent, Robinson (2023) [2] conceptualizes human translation to evolve on three levels of energetic, logical, and emotional interpretants (see Section 2). He explains the origins of the “logical interpretant” to emerge as a result of a clash between the emotional and the energetic interpretants. Conventionally, he says, theories assumed that natural language processing is “handled” entirely by logical interpretants, which is, in our architecture, a constitutive ingredient of the cognitive layer. The cognitive layer is, among other things, the location of conceptual planning, rational thought, and analytic thinking. For Robinson, however, cognition and the logical interpretant emerge in a process of “feeling-becoming-thinking”: “Whatever conscious awareness we have of semantic meaning is constantly being *fed* affectively, conatively, and becoming-cognitively to the logical interpretant by the emotional and energetic interpretants”. According to this view, processes on the cognitive layer would emerge when the coordination breaks down between the phenomenal and the sensorimotor layer, in an ”affective-becoming-cognitive trajectory”. 

This view has the advantage of rejecting—from the beginning—the existence of the “hard problem” of consciousness, as formulated by Chalmers (1996) [113]. Chalmers’s “hard problem” asks why humans have subjective experience (or phenomenal consciousness) at all, why physical processes are accompanied by an experiential aspect, when human behavior can be explained in mechanistic terms. It can be imagined (by some) that Zombies (that is, philosophical creatures that lack any kind of consciousness) are as successful as humans are. Under this assumption, Chalmers asked why we have feelings and emotions when they are not necessary to complete a task. However, Robinson’s notion of “feeling-becoming-thinking” alleviates this “hard problem” altogether, since his approach starts from first-person experience. Robinson takes “feeling-as-First”, rather than as a difficult-to-explain addition to an assumed functional human machine, the understanding of which constitutes Chalmers’s so-called “easy problem(s)”. 

Similarly, Seth (2021, p. 23) [18] formulates the “real problem of consciousness” that aims at explaining *why*—rather than establishing *that*—a neural, or, as in our case, a behavioral, pattern “maps to a particular kind of conscious experience”. Seth’s *real problem of consciousness* acknowledges the existence of phenomenal experiences and predicts that “the hard-problem intuition that consciousness can never be understood in physical terms will fade away, eventually vanishing in a puff of metaphysical smoke” (p. 28), since, as he posits at the end of his book “the entirety of human experience and mental life arises because of, and not in spite of, our nature as self-sustaining biological organisms that care about their own persistence.” (p. 266)

The effect of phenomenal experiences, Seth says, is a “massive reduction of uncertainty” (p. 53), as we can have at any one point in time only one conscious experience out of a vast possibility. However, conscious experience is more than just a reduction in uncertainty. Every conscious experience, Seth says, “is both informative and unified at the level of phenomenology”. (p. 54) Consequently, it can be assumed that a coherent interaction with the environment becomes difficult if experience disintegrates with sensorimotor processes. In such a coordination clash between the phenomenal layer (the emotional interpretant) and sensorimotor processes (the energetic interpretant), Robinson suggests that the logical interpretant (i.e., the cognitive layer) may then interfere to re-integrate sensorimotor contingencies with the phenomenal layer. 

If this is correct, Robinson’s view provides a complementary interpretation of our HOF taxonomy: as discussed in Section 4, a state of Hesitation is triggered in a moment of surprise or unanticipated observation and may lead to a temporary disruption/disintegration of the sensorimotor/phenomenologically integrated Flow states. In Robinson’s terminology, Hesitation states thus likely instantiate the “feeling-becoming-thinking” trajectory in which predominantly logical interpretants are “called upon to build an analytical bridge from source text to target language” (p. 38). An example of such an “analytical bridge” is provided in Section 4 when discussing the state of Hesitation in the translation of “*traditional*” and the syntactic shift it triggered. This is also corroborated in the HOF state transition Table 7 which shows that states of Hesitation are most frequently followed by a Flow state.

This view suggests that interventions of processes on the cognitive layer are triggered by a phenomenal disintegration as an effect of the “affective-becoming-cognitive trajectory”, rather than the other way around, in which Flow states would only be possible following a phase of cognitive/reflective intervention. However, whatever their order, there seems to exist an increasing agreement that Hesitation/extended reflection constitutes the exception rather than the “default”—at least for more experienced translators. PP and AIF may provide support for a deeper understanding of these intricate processes and their interactions.

Robinson also stipulates that the “affective-becoming-cognitive trajectory” would “explain conventionalization in all human social interaction, not just in language, […] conventionalization in linguistic usage and then, more narrowly, in translation” (p. 114), as discussed in the next subsection.

### 9.4. Translation Norms and the Extended Mind

Another related controversy touches the notion of “translation norms”. Drawing on Hermans, 2013 [114], Robinson, 2023, [2] (p. 94) defines translation norms as “shared expectations binding translators and their clients together”. Translation norms clarify “how one should translate: the best way to translate; the ideal” (ibid.) They determine “performance instructions” as well as “behavioural routines” that specify the type and extent of equivalence manifested by actual translations [13].

However, how these norms are established and what levels of mental processes they entertain is controversial. Chesterman, 1993 [115], for instance, defines translation norms as rational laws, clearly locating them on a cognitive layer. He says, “norms originate in rational, norm-directed strategies which are observed to be used by professionals. These laws are empirical, spatio-temporally falsifiable, probabilistic, predictive and explanatory”. 

This view has been contested by Robinson, 2023 [2] and others. Halverson and Kotze, 2022 [116], for instance, maintain that language use (and translation) is “convention- and entrenchment-driven” due to frequency effects. Through experience, translators have internalized much about which linguistic choices are considered appropriate in particular contexts and which are favored. In line with hypothesis 2 above, this view would hence designate the sensorimotor layer as the location of translation and translation norms. However, Halverson and Kotze underscore that “conventionalized norms also have a fully embodied and affective nature” (p. 71), which suggests that norms emerge as integrated processes on the sensorimotor and phenomenal layers. In addition, Halverson and Kotze also maintain that there is an “interwoven relationship between cognitive representations and social experience” (p. 73), which alludes to the cognitive layer. They conceptualize translation norms to emerge on two poles of a continuum, as “patterns of bottom-up conventionalization … and norms as socially constructed and legitimized collective agreements” (p. 68). Norms thus evolve, according to them, in a circular fashion, in terms of individual-internal and cumulative-collective versions that (presumably) approximate over time. Translation norms seem to transcend the translator’s physical boundaries and stretch into the social environment; the internal models become interwoven with collective agreements and social experience, touching all three processing layers of the translating agent. A translator then adjusts, over time, with collective agreements and intersubjective expectations, in line with a “conception of the agent as a model of its environment” ([107], p. 4807).

In the PP/AIF context, this process is known as “self-evidencing”: self-evidencing is a mechanism by which an agent gathers and samples sensory inputs that are already predicted and thus explained by the agent’s internal model. Self-evidencing, Kirchhoff and Kiverstein, 2021 [107] (p. 4808) say, is a process that contributes to maintaining the organization of the agent over time. It is “responsible for producing the boundary separating the agent from its surroundings”. However, according to Kirchhoff and Kiverstein, 2021 [107], this boundary is not fixed once and forever; the boundary of the mind “will coincide over time with the boundaries of the self-evidencing individual agent” (p. 4804). According to them, the agent–environment boundary marks the “causal-constitutive cut” which may change over time, as it specifies whether something is a component of a system (such as the mind) or merely an event (a cause) that leads to another event (the effect). 

Translation norms are thus constitutive of the translation process, rather than merely causal. They are constitutive of the translating mind, as they form prior beliefs that substantially reduce the translator’s prediction errors and they are—at least partially—outside the translator’s body. Translation norms, in this view, are examples of a “liberal view” of the extended mind hypothesis, which stipulates that cognition is “socially extensive, in a way that goes beyond the typical examples (involving notebooks and various technologies)” (Gallagher 2013, p. 1 [117]). The ‘classical’ Extended Mind Hypothesis, proposed by Andy Clark and David Chalmers, suggests that the mind is not confined to the brain or the body but extends into the environment through the use of external tools and technologies. Gallagher, 2013 [117] extends this classical notion, maintaining that the “socially extended mind is in some cases constituted not only in social interactions with others, but also in ways that involve institutional structures, norms, and practices.” (p. 1) Thinking, he says, cannot be reduced to purely “in the head” processes. Cognition is distributed: “There is a distribution across a number of participants—including the experts” (p. 4). 

A similar view can also be found in Toury, 2012 [13] (p. 64), who maintains that translation norms are negotiated “through a (largely unconscious) socialization and intersubjective alignment process in the course of which members of a community reach (mostly non-verbalized) agreements on appropriate behavior in particular contexts, based on general shared values”. In the same line of thought, Robinson, 2023 [2] highlights that translation is fundamentally “interactional in its very nature, involving—as any kind of interaction does—environmental feedback” (p. 284). We “turn repeated events into patterns that we take to be reality. Those patterns are norms” (2023, 94ff). It thus seems that, due to translation norms, translators can make sense of their observations in the translation job, as they would otherwise remain in a state of high prediction errors. Norms maximize evidence of the translator’s (expected) observations and—by adjusting their mental models accordingly—help them better predict their future (translational) action, thus reducing risks of surprise or hesitation, to better maintain a state of flow. 

Robinson (2023) further explains that norms “are ‘counterfactual’ orientations to action guided by shared regulatory social affect” (p. 101). Norms help us deal with the fear of failure for future action. According to Robinson, affective and emotional factors play a crucial role in norm formation. Much of our understanding (not only in translation) is related to “sensory-motor contingencies that afford us the ability to understand other people’s feelings and feeling-saturated thoughts”. He maintains that the formation of norms “may exist, be learnt and operate without ever being verbalized”. For Robinson, norms “rely on intuition or unconscious habit”, they are a “habit-as-instinct”. Similar to Halverson and Kotze’s “patterns of bottom-up conventionalization”, also Robinson explains that “we don’t so much register norms as create them, by way of making sense of events” (p. 110). He continues that “the counterfactual *fear of social disapproval* motivates the construction of norms *and* conformity to those newly constructed norms” (p. 110, original empathizes). Norms regulate “emotional response to events that have abnormal causes” (p. 103). Norms are thus instantiations of socially legitimized patterns that are vital for the self-evidencing agent [17], as they ensure the translator’s “systemic integration on average and over time” and to keep free energy (i.e., the amount of surprise and unexpected observations) to a minimum in the long run [118]. 

Kirchhoff and Kiverstein, 2021 [107] explain that “the mind is nested and multiscale sometimes extending beyond the individual agent to incorporate items located in the environment”. For them, “external resources form a part of an agent’s mind when they are poised to play a part in the processes of active inference that keep surprise to a minimum over time (i.e., that minimise free energy)” (p. 4807). The notion of a Markov Blanket (see Figure 4) is well suited to account for this nested conception of the mind. It implies that the mind has no unique, permanently fixed boundary: although the mind has only one boundary at any one point in time, “the Markov blanket concept can be put to work to delineate a boundary for the mind that changes over time to sometimes include elements external to the individual’s body” (ibid.). Extended minds incorporate tools, devices, routines and, as this discussion shows, also social practices and norms that “find their home in the surrounding environment”.

Intersubjective alignment processes and shared values across social groups may thus (temporally) extend the boundaries of the translator’s mind, as do translation aides and CAT tools [119]. The three layers of the proposed architecture and their predictions may then expand into that environment, taking as priors the anticipated expectations of external agents, customers, or the translation audience, by following or creating translation norms and producing “content according to agreed-upon specifications”.

## Figures and Tables

**Table 1:** Types of pauses in the TS Framework and some of their properties.

Type of Pause	Properties
Delay	IKI threshold: IKI ≥ 200 ms Separation between successive motor programs (3–4 keypresses) Motor programs are automatized typing routine (IKI < 200 ms)
Respite (*RSP*)	IKI threshold: RSP=2 ∗ median(within−word IKI) Separation between Tasks (sequences of motor programs) RSPs are non-intentional typing halts RSPs are part of fluent typing
Task Segment Pause (*TSP*)	IKI threshold: TSP=3∗median(between−word IKI) Separation between Task Segments (sequences of Tasks) *TSPs* are intentional typing halts *TSPs* disrupt the typing flow
Superpause	IKI threshold: Superpause >> TSP Superpauses separate Flow states Superpauses indicate first-pass readings or states of Orientation

**Table 2:** Three types of embedded processing layers of translation agent.

Type of Layer	Properties
Sensorimotor	Integration of sensory (textual) input and motor action (typing, Motor Programs) Short non-intentional breaks, facilitating fluent translation production Monitoring and adjustment through feedback loops Continuous interactions with the environment
Cognitive	Higher-order processes: reasoning, problem solving, memory, abstract thought Executive functions: attention, decision making, cognitive control, and planning Metacognition: reflection and evaluation, monitoring, inference, interpretation
Phenomenal	Subjective experiences, (self-) awareness, affect, emotions and feelings Experience of agency and volition Time perception, temporal order of events Sense of autonomy and responsibility for actions Self-reflection on desires, intentions, and beliefs

**Table 3:** The properties of the empirical keystroke data for from-scratch translation from English into 5 different languages. The “Study” (e.g., AR20) is the internal name in the CRITT TPR-DB and has no further meaning in this paper.

Study Name	AR20	BML12	KTHJ08	NJ12	SG12
Target Lang.	ar	es	da	hi	de
Keystrokes	37,171	73,619	72,383	43,137	58,883
Duration (h)	8.72	10.10	7.70	14.67	12.46
Sessions	40	60	69	38	47
Translators	22	32	24	20	24
Mean IKI	844	493	382	1223	761
Median IKI	265	156	160	374	156

**Table 4:** *RSP* and *TSP* values for 22 Arabic and 32 Spanish translators.

ar	Min	Max	Mean	Median
*RSP*	312	1032	563	546
*TSP*	795	2388	1288	1077
es				
*RSP*	220	470	301	281
*TSP*	423	1686	697	609

**Table 5:** The 11 most frequent types of *Task Segments* (TS label) and their percentages for the Spanish and Arabic translation data. The column “Total” shows the total number of *Task Segments*, and %es and %ar indicate the proportion of the TS label in the two languages. DurTS provides the average duration of the *Task Segment*. The Table shows the average IKI and the average number of keystrokes per *Task* (KeyT).

TS Label	Total	%es	%ar	DurTS	IKI	KeyT
**A**	3870	37.95	36.46	921	173	5.33
**AA**	1398	13.94	12.81	2167	211	5.13
**D**	753	7.71	6.59	504	121	4.16
**AAA**	543	5.49	4.86	2740	190	4.81
**AAAA**	263	2.72	2.25	4365	233	4.67
**DA**	194	2.15	1.44	1593	196	4.07
**AD**	164	1.35	1.96	1607	226	3.56
**C**	164	1.27	2.08	641	152	4.23
**DD**	116	1.42	0.64	1183	122	4.84
**AAAAA**	107	1.06	0.99	5300	230	4.61
**CC**	84	0.62	1.11	1181	160	3.70

**Table 6:** The number of HOF translation states in the manually annotated Spanish and Arabic data and respective percentages. There are approximately half the number of states for Arabic for 25% less annotated data, but the ratio of the three states seems to be quite similar for the two language pairs.

	O	%O	F	%F	H	%H	Total
es	183	30.20	284	46.86	139	22.94	606
ar	93	31.85	132	45.21	67	22.95	292

**Table 7:** Transition matrix between successive HOF states for Arabic (left) and Spanish (right).

		ar			es		
	To	O	F	H	O	F	H
From	O	-	**0.84**	0.16	-	**0.86**	0.14
	F	**0.60**	-	0.40	**0.60**	-	0.40
	H	0.21	**0.79**	-	0.09	**0.91**	-

**Table 8:** The proportions of Tasks in Flow and Hesitation states for the Spanish and Arabic data. There is clearly a higher proportion of **A** Tasks in Flow states (84% and 81%) and more **D** Tasks during Hesitation. (Columns add up to 100%.)

	ar		es	
	H	F	H	F
**A**	0.54	0.84	0.53	0.81
**D**	0.34	0.08	0.41	0.08
**C**	0.12	0.08	0.06	0.11

**Table 9:** The six most frequent Task Segment labels of Flow and Hesitation states in Arabic and Spanish. Note the identical ranking of Task Segment labels in the Flow state (F-ar and F-es).

F-ar	F-es	H-ar	H-es
**A**	**A**	**A**	**A**
**AA**	**AA**	**D**	**D**
**AAA**	**AAA**	**AA**	**C**
**AAAA**	**AAAA**	**C**	**AA**
**C**	**C**	**DD**	**DA**
**D**	**D**	**DA**	**CA**

**Table 10:** Summary information of Flow states for Arabic and Spanish data. Mean, minimum, and maximum values per translation session: average duration (Dur/F), keystrokes (Key/F), Task Segments (TS/F), and Tasks (T/F) per Flow state, keystrokes per Task Segment (Key/TS), and keystrokes per Task (Key/T).

ar	Dur/F	Key/F	TS/F	Key/TS	T/F	Key/T
mean	12320	36.58	3.04	12.50	7.17	5.04
min	8314	23.48	1.48	10.67	5.22	4.10
max	21930	63.33	5.60	15.88	10.40	6.09
**es**						
mean	6389	29.42	2.10	14.85	5.44	5.47
min	3585	19.52	1.30	10.76	4.00	4.69
max	9484	40.97	3.81	20.04	7.80	6.70

**Table 11:** Summary information of Hesitation states for Arabic and Spanish data. Same columns as in Table 10.

ar	Dur/H	Key/H	TS/H	Key/TS	T/H	Key/T
mean	14,155	17.68	2.76	6.78	4.54	3.99
min	9703	11.15	2.14	3.82	3.62	2.51
max	17,597	24.14	3.38	11.27	6.26	6.26
**es**						
mean	7329	3.19	1.95	1.55	2.23	1.34
min	4199	1.71	1.53	1.07	1.55	1.02
max	13,122	7.64	2.59	2.95	3.32	2.30

**Table 12:** Duration of Orientation states, Arabic and Spanish translations.

	Mean	Min	Max
ar	10,300	5851	15,169
es	4838	3007	8050

## Data Availability

The data used in this article are freely available and can be downloaded from the CRITT website. The CRITT provides free server access through registration via: https://sites.google.com/site/centretranslationinnovation/tpr-db/getting-started (accessed 12 July 2024). Upon logging into the CRITT server as *summer_gst*, a Python notebook is available under *shared/IKI_analysis.ipynb* that contains the Python code and data used in Section 7 and Section 8 of this study.
